# Knowledge, Attitude, and Practice of Diagnostic Radiology Among Clinical Year Medical Students

**DOI:** 10.7759/cureus.58624

**Published:** 2024-04-20

**Authors:** Reem Brashi, Basem Bahakeem, Shumok S Almatrfi, Sara B Badirah, Muhjah M Almurakshi, Bayan F Hafiz, Ayman Eskandar, Turki Alhazmi, Shakeeb Irfan, M. Irfanullah Siddiqui

**Affiliations:** 1 College of Medicine and Surgery, Umm Al-Qura University, Makkah, SAU; 2 College of Medicine and Surgery, Department of Medical Imaging, Umm Al-Qura University, Makkah, SAU; 3 College of Medicine and Surgery, Department of Internal Medicine, Creek General Hospital, Karachi, PAK

**Keywords:** college, medical student, practice, knowledge, radiology

## Abstract

Background

Nowadays, radiology is considered one of the most important disciplines of medicine as it guides physicians to reach the proper diagnosis by using many types of medical imaging modalities, such as x-ray radiography, computed tomography (CT), ultrasonography (US), and magnetic resonance imaging (MRI). These modalities are used to create dynamic images of different parts of the human body, which are being used to accurately diagnose and follow up on a variety of medical conditions. Moreover, in recent decades, radiology has experienced substantial growth and transformation, establishing itself not just in diagnostics but also in the domain of medical interventions, which includes the increasingly recognized discipline of interventional radiology.

Methodology

A descriptive cross-sectional study was conducted at Umm Al-Qura University (UQU) in Makkah from October 2022 to April 2023. The population size of medical students from the fourth to sixth year at Umm Al-Qura University is about 820 students.

Results

The total number of participants was 359, but two did not fill out properly. Hence, data was analyzed for (n=357), whereas more than half of them were female, 195 (54.6%). About 133 (37%) of the students were in their sixth year, while 106 (29%) were in their fourth year. Regarding their grade point average (GPA), 209 (58%) had >3.5, and 119 (33%) had 3.0-3.5. The maximum number of females 77 (57.9%) students were in the sixth year. Regarding knowledge, 291 (81.5%) had heard about interventional radiology before, while 66 (18.5%) had never heard about interventional radiology before. Moreover, 270 (75.6%) believe that the years in radiology residency are the same as other specialties. Regarding perceived knowledge about radiology, 183 (51.3%) said it is adequate.

Conclusion

This study showed that medical students at Umm Al-Qura University have a positive attitude towards radiology. However, the majority of the students do not have adequate knowledge regarding radiology as well as radiation hazards. We can improve this by increasing radiology experience in our institutions. Also, courses should be designed and incorporated into the curriculum to increase the knowledge of medical students about ionizing radiation.

## Introduction

Radiology is a critical branch of medicine, playing a pivotal role in aiding physicians to make accurate diagnoses. Various technological modalities, such as x-ray radiography, computed tomography (CT), ultrasonography (US), fluoroscopy, and magnetic resonance imaging (MRI), can produce images of the human body for accurate diagnosis and monitor various medical conditions [1]. In recent years, there has been a significant development in radiology with the emergence of interventional radiology (IR) [2]. Despite its significance, radiology has been fully integrated into teaching and training curriculums. Therefore, radiology is not exclusive to radiologists or students pursuing radiology; it encompasses other specialties such as internal medicine, obstetrics and gynecology, general surgery, and emergency departments [3,4].

Notably, the number of radiologists and various medical imaging modalities is much lower in primary healthcare centers than in central hospitals, making it challenging for non-radiological healthcare practitioners to interpret radiological studies. Therefore, building a solid foundation of knowledge from the preclinical years is vital as it can help improve their radiological image interpretation skills in the future [5]. Incorporating radiology into teaching and training curriculums is also crucial to equip students with fundamental knowledge about the imaging of normal body anatomy, standard radiological features, and appropriate studies for evaluating common pathologies and life-threatening conditions. It also helps prevent management failure and adverse outcomes, improving patient care [2,6].

Various studies have confirmed that a good teaching curriculum and a high amount of medical students’ exposure to radiology have a statistically significant impact on choosing radiology as an elective or a career choice in radiology. For instance, Pennsylvania has introduced students to a new curriculum, giving them great exposure to radiology. The results showed that 73% found radiology interesting, and 84% considered it a career [4]. Students at Indiana University School of Medicine have also completed a comprehensive radiology training program, increasing applications to the radiology residency program [7]. Another study conducted among foundation doctors in the UK revealed that 69% of the participants felt that their medical school training prepared them effectively to interpret essential imaging in the emergency setting, and 77% wished to have more radiology training [8].

Umm Al-Qura University (UQU) has recently integrated the radiology teaching curriculum with other subjects, and this modification aims to improve students' understanding of medical images, exposing them to radiology to change their opinion. This study assesses diagnostic radiology knowledge, attitudes, and practice among the medical students at UQU, Makkah, Saudi Arabia. The study also examines their opinion towards the current teaching curriculum and how it affects their judgment on choosing radiology as a future career. 

## Materials and methods

This descriptive cross-sectional study was conducted between October 2022 and April 2023 at UQU in Makkah. The study included 820 medical students in their fourth, fifth, and sixth clinical years at UQM. However, students previously attending imaging courses at UQU or outside were excluded from the study.

The study uses the snowball sampling technique to determine the sample size. The sample size was determined using the Epi Info™ program version 3.0 (CDC, Atlanta, GA, USA, 2011). A minimum of 367 participants were calculated using a snowball sampling technique. Moreover, the confidence interval (CI) level was set at 95%; an expected frequency percentage level was 50%, and a design effect was set to 1. The sample size was calculated to be 367 participants. The final sample size was 359. Out of this data, 357 were analyzed due to incomplete responses from two students.

For data collection, the study used an online, modified, valid English questionnaire designed by Google Forms, which was modified to be easily understood and distributed electronically via social media. It consisted of 36 concise, closed, and open-ended questions. The first section included questions about the participant's demographic data, including age, study year, grade point average (GPA), which was calculated on a scale ranging from 0 to 4 in UQU, attitudes towards radiology as a future career, background, and experience. The second section consisted of items to assess participants' knowledge and practice regarding radiology and radiation hazards. Participants were given eight radiological images of various health disorders and asked to choose the correct answers. A radiology specialist was selected to determine the right answer. The third section of the questionnaire focused on assessing the source of information and gathering opinions about the radiology curriculum. The questions included 11 knowledge questions, six attitude questions, and eight practice questions. Any correct answer or positive attitude received a score of "1" and a "0" for any wrong answer. Participants who answered less than 50% of the questions correctly were considered to have a poor knowledge level; those who answered between 50% correctly were supposed to have a fair knowledge level, and those who responded 75% correctly were considered to have a good knowledge level. Attitude and practice levels were scored similarly.

The data was statistically analyzed using SPSS version 20.0 (IBM, New York, USA) [9]. Qualitative data was presented as numbers and percentages to test the relationship between variables, and the Chi-squared test (χ^2^) was used to find differences in various groups. However, quantitative data were expressed as mean and standard deviation (mean ± SD). Correlation analysis was conducted using Spearman's test, and statistical significance was defined as a p-value <0.05.

## Results

In the study of 357 students, 54.6% were females, while 37.3% were in the sixth academic year. The majority of the students had a GPA of >3.5 (58.5%) (Table 1). 

**Table 1:** Distribution of the participants according to their demographic and academic data (N: 357). GPA: grade point average.

Variable	N (%)
Gender	
Female	195 (54.6)
Male	162 (45.4)
Academic year	
Fourth	106 (29.7)
Fifth	118 (33.1)
Sixth	133 (37.3)
GPA	
<2.5	3 (0.8)
2.5-2.9	26 (7.3)
3.0-3.5	119 (33.3)
>3.5	209 (58.5)

Moreover, most students reported that the radiology residency program had the same years as other specialties (75.6%), and 81% reported having prior knowledge of IR. Regarding students' knowledge, the majority (94.1%) knew that radiation could affect pregnancy, 77.3% knew that the first trimester of pregnancy was more sensitive to radiation, and 89.1% knew the presence of a specific limit of radiation exposure for patients per year.

Table 2 shows that 38.9% mentioned that ultrasonography is a radiological modality having no radiation, and 48.2% reported that the testis and ovaries are the most sensitive organs to radiation. 

**Table 2:** Distribution of the participants' responses regarding their knowledge about radiology (N: 357). CT: computed tomography; MRI: magnetic resonance imaging; US: ultrasound.

Variable	N (%)
How many years is the radiology residency program in comparison with other specialties?	
Much less	74 (20.7)
Much more	13 (3.6)
The same	270 (75.6)
Have you ever heard of interventional radiology before?	
No	66 (12.5)
Yes	291 (81.5)
Does the radiation affect the pregnancy?	
No	21 (5.9)
Yes	336 (94.1)
Which trimester of pregnancy is more sensitive to radiation?	
First trimester	276 (77.3)
Second trimester	57 (16)
Third trimester	24 (6.7)
Is there a specific limit of radiation exposure for patients per year?	
No	39 (10.9)
Yes	318 (89.1)
Which radiological modality has no radiation?	
MRI	55 (15.4)
US	139 (38.9)
US, MRI	108 (30.3)
X-ray	22 (6.2)
X-ray and/or CT	32 (9)
None	1 (0.3)
Which are the most sensitive organs to radiation?	
Bone	1 (0.3)
Breast	69 (19.3)
Liver, bladder, kidney	75 (21)
Lungs, colon	35 (9.8)
Testis and ovaries	172 (48.2)
Thyroid gland	2 (0.6)
I don’t know	3 (0.9)

Most students (73.1%) mentioned that mammography is the radiological modality used to screen breast cancer. However, 48.7% of students reported that CT was the radiological modality used for screening abdominal aortic aneurysms. About 40.1% mentioned that a bone density scan, abbreviated as dual-energy x-ray absorptiometry (DEXA), is the radiological modality for screening osteoporosis. However, 44% mentioned that CT or X-ray is the radiological modality to screen lung cancer (Table 3).

**Table 3:** Distribution of the participants' responses regarding their knowledge of radiological screening for different health disorders (N: 357). CT: computed tomography, DEXA: dual-energy x-ray absorptiometry, US: ultrasound.

Variable	CT	DEXA	Mammography	US	X-ray	None
Which radiological modality could be used for screening of:						
Breast cancer	12 (3.4)	1 (0.3)	261 (73.1)	55 (15.4)	23 (6.4)	5 (1.4)
Abdominal aortic aneurysm	174 (48.7)	10 (2.8)	8 (2.2)	91 (25.5)	65 (18.2)	9 (2.5)
Osteoporosis	38 (10.6)	143 (40.1)	10 (2.8)	14 (3.9)	143 (40.1)	9 (2.5)
Lung cancer	157 (44)	9 (2.5)	8 (2.2)	8 (5)	157 (44)	8 (2.2)

Radiology is an exciting field. The findings indicate that 72.5% of students are interested in learning more about this field, with over 58% considering taking one radiology elective course. In comparison, 13.7% are willing to take several courses in radiology. Furthermore, 51.3% of students believed that their knowledge of radiology was adequate compared to the other fields, demonstrating a solid foundation for further learning. Radiology fits the lifestyle of 61.6% of students; 53.8% consider diagnostic radiology a future career, and 52.1% positively reported that radiology impacts diagnosing as a critical physical exam process (Table 4).

**Table 4:** Participants' attitude towards radiology specialty (N: 357).

Variable	N (%)
Attitude	
Are you interested in learning more about this field?	
No	98 (27.5)
Yes	259 (72.5)
Would you like to take a radiology elective course?	
Maybe one radiology elective	207 (58)
Not a chance	101 (28.5)
Probably several radiology electives	49 (13.7)
How would you rate your knowledge about radiology compared to the other fields?	
Adequate	183 (51.3)
Excellent	10 (2.8)
Good	64 (17.9)
Poor	100 (28)
Do you think the lifestyle in radiology fits you?	
No	137 (38.4)
Yes	220 (61.6)
Would you consider diagnostic radiology as a future career?	
No	165 (46.2)
Yes	192 (53.8)
How much of an impact does radiology have on the diagnosing process?	
As important as the physical exam	186 (52.1)
Minimal impact	18 (5)
More important than a physical exam	43 (12)
Occasionally changes patient care	36 (10.1)
Often changes patient care	74 (20.7)

**Figure 1:**
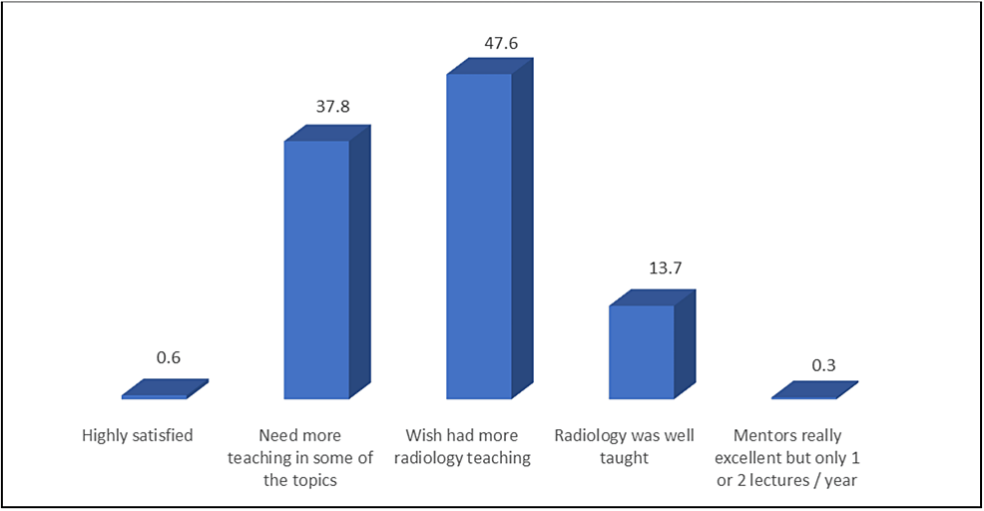
Percentage distribution of the students' attitude towards the radiology teaching curriculum in Umm Al-Qura University (N: 357).

When assessing students' attitudes towards the radiology teaching curriculum in UQU, 47.6% of students expressed a strong desire for more radiology teaching, and 37.8% mentioned that certain areas within the curriculum require further teaching, as depicted in Figure 1.

**Figure 2:**
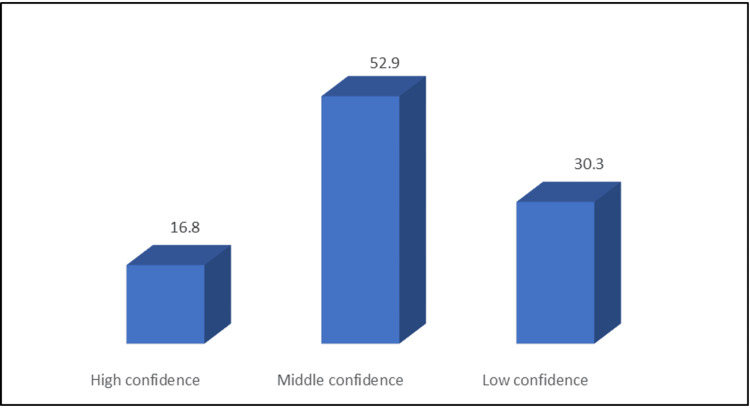
Percentage distribution of participants' rating of their confidence in diagnosing the provided eight images (N: 357).

The students were provided eight radiological images to assess their practice in accurately diagnosing these images. Out of these, 59.7% of students correctly diagnosed five pneumoperitoneum images. As presented in Table 5, 55.5% correctly diagnosed large bowel obstruction, epidural hemorrhage (58.3%), cholecystitis (54.6%), and hydronephrosis (62.5%). Only 16.8% were highly confident in diagnosing the previous images, while 52.95% had moderate confidence (Figure 2).

**Table 5:** Participants' practice in diagnosing of different radiological images (N: 357).

Image	Diagnosis	Correct answers N (%)
1	Pneumoperitoneum	213 (59.7)
2	Tension pneumothorax	164 (45.9)
3	Large bowel obstruction	198 (55.5)
4	Subarachnoid hemorrhage	173 (48.5)
5	Epidural hemorrhage	208 (58.3)
6	Renal mass	130 (36.4)
7	Cholecystitis	195 (54.6)
8	Hydronephrosis	223 (62.5)

**Figure 3:**
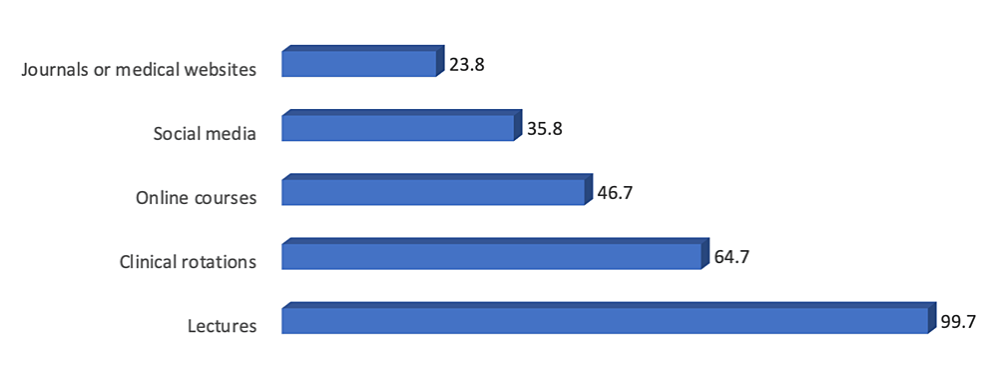
Sources of radiology information among studied students (N: 357).

Most students rely on lectures (99.7%) and clinical rotations (64.7%) as the primary sources of radiology information (Figure 3).

Participants suggested some solutions to improve the radiology teaching curriculum. These solutions include case-based learning for discussing different cases and types of radiological modalities and a systematic approach to diagnosing every imaging modality. Other solutions are to increase radiological lectures and training at all educational levels, provide specialized staff with clear, informative lectures, and offer training rotations on case slides and workshops.

**Figure 4:**
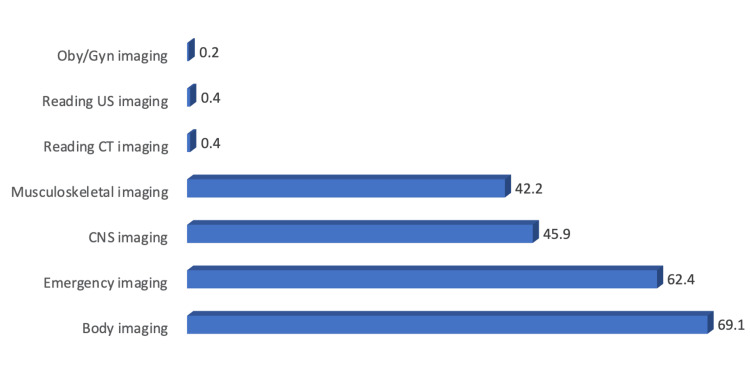
Radiological topics needed to be taught more according to students' point of view (N: 357). CT: computed tomography, US: ultrasound, OBY/GYN: obstetrics and gynecology, CNS: central nervous system.

The study found that the most common topics that need more teaching, according to students, were body imaging (69.1%) and emergency imaging (62.4%) (Figure 4).

**Figure 5:**
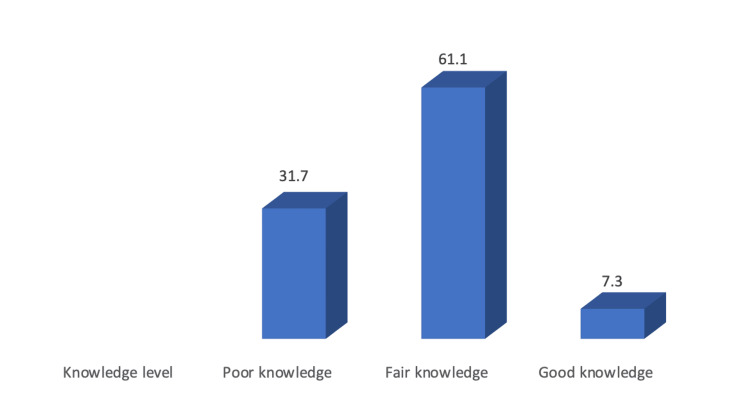
Participants' level of knowledge about radiology (N: 357).

**Figure 6:**
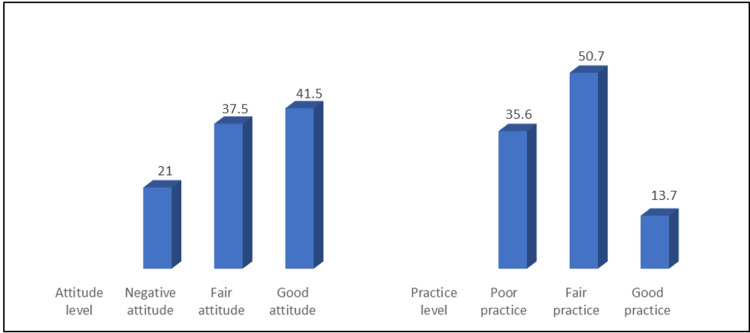
Levels of attitude and practice regarding radiology (N: 357).

Figures 5, 6 show that only 7.3% of students have a good knowledge of radiology, while 61.1% have failed knowledge about radiology. In addition, 41.5% had a positive attitude, and only 13.6% had a good practice level.

The study found that the mean knowledge score among students was 6.06 ± 1.83, while the mean total attitude and practice scores were 3.83 ± 1.6 and 4.21 ± 1.92, respectively. Table 6 demonstrates a significant correlation between high levels of knowledge and students with the highest GPA (>3.5), as well as those expressing low confidence in diagnosing the given images (p = <0.05).

**Table 6:** Relationship between students' knowledge level about radiology and their demographic and academic data, perception of the radiology teaching curriculum in Umm Al-Qura University and confidence in diagnosing of the provided eight radiological images (N: 357). GPA: grade point average.

Variable	Knowledge level			χ^2^	p-value	
	Poor	Fair	Good			
	N (%)	N (%)	N (%)			
Gender						
Female	52 (46)	127 (58.3)	16 (61.5)	5.03		0.08
Male	61 (54)	91 (41.7)	10 (38.5)			
Academic year						
Fourth	42 (37.2)	59 (27.1)	5 (19.2)	5.42		0.246
Fifth	35 (31)	74 (33.9)	9 (34.6)			
Sixth	36 (31.7)	85 (39)	12 (46.2)			
GPA						
<2.5	0 (0.0)	3 (1.4)	0 (0.0)	29.29		<0.001
2.5-2.9	19 (16.8)	4 (1.8)	3 (11.5)			
3.0-3.5	40 (35.4)	72 (33)	7 (26.9)			
>3.5	54 (47.8)	139 (63.8)	16 (61.5)			
How do you feel regarding the radiology teaching curriculum in Umm Al-Qura University?						
Highly satisfied	0 (0.0)	2 (0.9)	0 (0.0)	4.86		0.722
I need more teaching on some of the topics	39 (34.5)	84 (38.5)	12 (46.2)			
I wish that I had more radiology teaching	57 (50.4)	104 (47.7)	9 (34.6)			
Radiology has been well-taught	17 (15)	27 (12.4)	5 (19.2)			
The mentors are really excellent but only one or two lectures per year	0 (0.0)	1 (0.5)	0 (0.0)			
Participants' rating of their confidence in diagnosing the previous pictures						
High confidence	10 (8.8)	43 (19.7)	7 (26.9)	12.45		0.014
Middle confidence	45 (39.8)	58 (26.6)	5 (19.2)			
Low confidence	58 (51.3)	117 (53.7)	14 (53.8)			

However, no significant relationship was found between students' attitudes towards radiology and their demographic and academic data, perception of the radiology teaching curriculum in UQU, and confidence in diagnosing the eight radiological images (p = >0.05), as shown in Table 7.

**Table 7:** Relationship between students' attitude level towards radiology and their demographic and academic data, perception of the radiology teaching curriculum in Umm Al-Qura University and confidence in diagnosing of the provided eight radiological images (N: 357). GPA: grade point average.

Variable	Attitude level			χ^2^	p-value	
	Poor	Fair	Good			
	N (%)	N (%)	N (%)			
Gender						
Female	43 (57.3)	72 (53.7)	80 (54.1)	0.28		0.867
Male	32 (42.7)	62 (46.3)	68 (45.9)			
Academic year						
Fourth	22 (29.3)	38 (28.4)	46 (31.1)	2.06		0.725
Fifth	21 (28)	49 (36.6)	48 (32.4)			
Sixth	32 (42.7)	47 (35.1)	54 (36.5)			
GPA						
<2.5	0 (0.0)	1 (0.7)	2 (1.4)	3.5		0.741
2.5-2.9	5 (6.7)	13 (9.7)	8 (5.4)			
3.0-3.5	23 (30.7)	45 (33.6)	51 (34.5)			
>3.5	47 (62.7)	75 (56)	87 (58.8)			
How do you feel regarding the radiology teaching curriculum in Umm Al-Qura University?						
Highly satisfied	0 (0.0)	0 (0.0)	2 (1.4)	9.53		0.299
I need more teaching on some of the topics	35 (46.7)	45 (33.6)	55 (37.2)			
I wish that I had more radiology teaching	29 (38.7)	72 (53.7)	69 (46.6)			
Radiology has been well-taught	11 (14.7)	16 (11.9)	22 (14.9)			
The mentors are really excellent but only one or two lectures per year	0 (0.0)	1 (0.7)	0 (0.0)			
Participants' rating of their confidence in diagnosing the previous pictures						
High confidence	13 (17.3)	18 (13.4)	29 (19.6)	2.28		0.684
Middle confidence	24 (32)	43 (32.1)	41 (27.7)			
Low confidence	38 (50.7)	73 (54.5)	78 (52.7)			

On the other hand, Table 8 illustrates that good practice is significantly higher among female students and those with the highest GPA (>3.5). The table also suggests that students who express low confidence in diagnosing the images are likely to have a high GPA (p = <0.05).

**Table 8:** Relationship between students' practice level regarding radiology and their demographic and academic data, perception of the radiology teaching curriculum in Umm Al-Qura University and confidence in diagnosing of the provided eight radiological images (N: 357). GPA: grade point average.

Variable	Practice level			χ^2^	p-value	
	Poor	Fair	Good			
	N (%)	N (%)	N (%)			
Gender						
Female	49 (38.6)	108 (59.7)	38 (77.6)	25.43		<0.001
Male	78 (61.4)	73 (40.3)	11 (22.4)			
Academic year						
Fourth	48 (37.8)	47 (26)	11 (22.4)	6.7		0.153
Fifth	36 (28.3)	65 (35.9)	17 (34.7)			
Sixth	43 (33.9)	69 (38.1)	21 (42.9)			
GPA						
<2.5	1 (0.8)	2 (1.1)	0 (0.0)	19.22		0.004
2.5-2.9	17 (13.4)	9 (5)	0 (0.0)			
3.0-3.5	50 (39.4)	54 (29.8)	15 (30.6)			
>3.5	59 (46.5)	116 (64.1)	34 (69.4)			
How do you feel regarding the radiology teaching curriculum in Umm Al-Qura University?						
Highly satisfied	0 (0.0)	0 (0.0)	2 (4.1)	19.72		0.011
I need more teaching on some of the topics	47 (37)	71 (39.2)	17 (34.7)			
I wish that I had more radiology teaching	61 (48)	85 (47)	24 (49)			
Radiology has been well-taught	19 (15)	25 (13.8)	5 (10.2)			
The mentors are really excellent but only one or two lectures per year	0 (0.0)	0 (0.0)	1 (2)			
Participants' rating of their confidence in diagnosing the previous pictures						
High confidence	9 (7.1)	30 (16.6)	21 (42.9)	37.82		<0.001
Middle confidence	52 (40.9)	49 (27.1)	7 (14.3)			
Low confidence	66 (52)	102 (56.4)	21 (42.9)			

A significant positive correlation was found between knowledge scores and attitude scores (r = 0.1, p-value = 0.039), and between knowledge scores and practice scores (r = 0.37, p-value = <0.001). While a non-significant positive correlation was found between attitude scores and practice scores (r = 0.1, p-value = 0.053).

As shown in Table 9, we observed a significant correlation between students who have a positive attitude toward radiology as a specialty and those willing to consider diagnostic radiology as a future career. The percentage of those with a positive attitude towards the radiology specialty is significantly higher among those willing to consider diagnostic radiology as a future career (p = −<0.05).

**Table 9:** Relationship between the students' willingness to consider diagnostic radiology as a future career and their knowledge, attitude, and practice levels regarding radiology.

Variable	Willing to consider diagnostic radiology as a future career		χ^2^	p-value
	Not willing N (%)	Willing N (%)		
Knowledge level				
Poor knowledge	50 (30.3)	63 (32.8)	0.35	0.837
Fair knowledge	102 (61.8)	116 (60.4)		
Good knowledge	13 (7.9)	13 (6.8)		
Attitude level				
Negative attitude	70 (42.4)	5 (2.6)	18.02	<0.001
Fair attitude	87 (52.7)	47 (24.5)		
Positive attitude	8 (4.8)	140 (72.9)		
Practice level				
Poor practice	59 (35.8)	68 (35.4)	0.7	0.705
Fair practice	86 (52.1)	95 (49.5)		
Good practice	20 (12.1)	29 (15.1)		

## Discussion

Radiology plays a critical role in helping physicians make accurate diagnoses, which is why it is considered one of the most essential disciplines of medicine. This study assessed medical students' knowledge, attitudes, and practice of diagnostic radiology at UQU. The study also sought students' opinions regarding the current teaching curriculum and how it influences their decision to pursue radiology as a future career.

The study found that over half of the participants, which was 186 (52.1%), believed that radiology was as crucial as physical examination. This result revealed a lower percentage of medical students who thought radiology was as vital as physical examination compared to a study conducted in Syria by Alchallah, who found that 199 (74%) were critical to physical examination [2]. One possible explanation for the difference in results could be the difference in population size. Understandably, the results vary as students learn about radiology images, primarily through lectures designed to help them understand how these images can assist with disease identification.

The study also found that only 64 (17.9%) students rated their perceived radiology knowledge as good, lower than that of the Alchallah study, which was 91 (33.8%). This difference could be due to the limited exposure to radiology during college, as their learning was predominantly lecture-based. When assessing interventional radiology knowledge (IR), 291 (81.5%) had heard about IR before. The results were better than those of the Alchallah study, where more than half of the population had heard about IR. These results indicate an increased awareness of IR [2]. 

This difference could be due to the limited exposure to radiology during college, as their learning was predominantly lecture-based. When assessing interventional radiology knowledge (IR), 291 (81.5%) had heard about IR before. The results were better than the previously published studies, as 63% of students in a European country rated their knowledge of IR as poor, as did the Alchallah study, where more than half of the population had heard about IR. These results indicate an increased awareness of IR among UQU medical students [2,9].

Our results are encouraging, as most students are aware of the effects of radiation on pregnancy (94.1%). These findings were consistent with previous studies conducted on Syrians (97.4%) and Nigerians (89.9%), highlighting the importance of this knowledge in medical education [2,6].

Furthermore, 89% of students in this study and those in the Norwegian study were aware of the specific limit of radiation exposure permitted for patients each year, implying that the medical examination involving radiation was justified and necessary for a clinical diagnosis [10]. This finding highlights the importance of prioritizing patient safety and minimizing unnecessary radiation exposure by healthcare professionals.

The International Committee on Radiological Protection 2007 has identified the ovaries, testes, bone marrow, and eye lens as the most radiosensitive organs to radiation exposure [2]. However, only 48.2% of students in the current study mentioned the testes and ovaries as the most sensitive organs to radiation. This result contradicted the previous literature, as in Australian and Syrian studies, students had better knowledge, as 83% and 65.1%, respectively, responded correctly [2,11].

Regarding students' knowledge about the radiation hazards of the modality, only 38.9% of medical students think that ultrasonography has lower radiation than other modalities. Additionally, 30.1% believe that ultrasonography and MRI have less radiation. A similar study found that only 42% of medical students identified ultrasound as the safest imaging method [2]. This lack of knowledge regarding using non-ionizing radiation in MRI and ultrasonography could lead to inappropriate imaging being ordered, especially if physicians are unaware that MRI and ultrasonography use non-ionizing radiation [10].

We also evaluated the student's knowledge about applying radiological screening for life-threatening and non-life-threatening conditions. Of those surveyed, 261 (73.1%) correctly identified mammography for breast cancer, 143 (40.1%) selected DEXA for osteoporosis, 157 (44.0%) selected CT for lung cancer, and 91 (25.5%) selected ultrasonography correctly for abdominal aortic aneurysm.

Overall, our study had a lower level of knowledge than the Alchallah study. The Alchallah study correctly identified mammography for breast cancer detection (n = 262 (97.4%)), DEXA for osteoporosis detection (n = 212 (78.8%)), CT for lung cancer detection (n = 206 (76.6%)), and ultrasound for abdominal aortic aneurysm detection (n = 186 (69.1%)). These results could also be explained by a lack of exposure to radiology and a small population size [2]. We found that students with GPA >3.5 had a significantly higher level of knowledge. However, good practice was substantially higher among female students, those with a GPA >3.5, those who wished to have more radiology teaching, and those who rated their confidence in diagnosing images as high. Remarkably, no significant gender difference was observed in knowledge and attitude scores [2].

We found significant differences among males and females concerning diagnostic skill practice and total scores, with a p-value of <0.05. A study on Syrian students reported comparable knowledge scores between males and females with no significant difference, as reported by Alchallah et al [2]. Similar findings were reported by Alnajjar et al. [12] regarding our result, as the curriculum is the same for both genders. We believe it may be affected by the difference in the number of female students who responded to the questionnaire.

Regarding self-confidence in diagnosing the eight images, only 16.8% of participants were highly confident in their answers, which was lower compared to the Samara study, where 63.8% reported a high level of confidence when diagnosing a life-threatening condition with no statistically significant difference based on GPA or gender [13].

Interestingly, in a recent study, lectures were found to be the primary source of radiological information for students, with a whopping 99.7% of participants relying on them. Clinical rotations were also popular, with 64.7% of students citing them as their to go resource. Interestingly, this differs from a previous study of Syrian medical undergraduates, where the Internet (33.5%) and social media (30.5%) were the most common sources of radiology knowledge. These differences in attitudes and preferences towards learning methods may be a factor in the varying results [2].

Interestingly, most students (47.6%) have expressed the need for more teaching in radiology. Similar results were presented by a study conducted among final-year medical students of the Faculty of Medicine, University of Jos, Nigeria. This study was consistent with the results presented by a similar study, which found that 47.6% of the participants expressed that the teaching method was inadequate. According to medical students at the University of Calabar, Nigeria, radiology and anatomy should be combined in preclinical courses, as 69.6% strongly agreed or agreed with the idea [6,14]. In addition, the medical students in the Visscher study expressed similar concerns and recommended enhancing the quality of radiology education through both formal and informal exposure [15].

Radiology is a field that has captured the interest of many students as a future career. As a result, 192 (53.8%) students considered diagnostic radiology their preferred specialty for future careers. This percentage was significantly higher than the percentages reported by the Alchallah study, where only 24.5% considered radiology a future career. Similarly, Vinod et al. found that 27% of students were interested in specializing in radiology because of its impact on patient care. In a study conducted in Nigeria, Salaam et al. found that only 21.8% showed interest in radiology as a career, and the male students showed statistically significant interest in the field compared to female students [1,2,14].

Notably, 58% of students in the current study chose radiology as an elective training option, higher than the 22.6% reported in Vinod et al.'s study [1]. These findings indicate that radiology is a popular and growing field among students. Therefore, students who are willing to consider diagnostic radiology as a future career tend to have a more positive attitude toward the radiology specialty. As a result, providing the students with more information regarding the specialty is crucial to helping them make informed decisions. By doing so, we can positively influence students' willingness to apply for radiology residency programs in Saudi Arabia [1,2,14].

The present study has discovered a significant positive correlation between knowledge scores and both attitude and practice scores. In a previous study, students who had undertaken the revised curriculum with more didactic radiology teaching were compared with students who had undertaken the traditional curriculum. The study found that early exposure to radiology positively affected students' future plans. Moreover, the number of students considering clinical radiology as an elective increased from 67% to 84%. In contrast, those who considered radiology as a career increased from 38% to 54% [4]. Therefore, it is imperative to consider curriculum changes for early exposure to radiology to raise the students' awareness and attitude towards the radiology specialty and encourage them to choose it as a future career.

This study has some strengths in being the first to be conducted in Saudi Arabia. Another significant aspect was the participation of students from all clinical years, including fourth, fifth, and sixth years, which allowed evaluation of the quality of the radiology educational process and the adequate inclusion of radiology in the curriculum and students' exposure in clinical years. This evaluation will lead to recommendations for improvement.

This study has some limitations. First, participants were enrolled at a single Saudi university and may not reflect the entire student population in Saudi Arabia. Similar studies could be conducted nationally to better understand the topic. Second, self-administrative surveys may be subject to some degree of response bias, as participants interested in the topic may be more likely to complete them.

## Conclusions

This study showed that medical students at UQU have a positive attitude toward radiology despite inadequate knowledge and practice. However, 53.8% considered diagnostic radiology as their future career. Almost half of the students (47.6%) desired that more radiology lectures be added to the curriculum, showing a high interest in learning radiology. However, the student showed lower knowledge about the radiation effect compared to other studies. As a result, some curriculum changes are needed to provide early exposure to radiology. This exposure could help raise students' awareness and attitude towards the radiology specialty and perhaps even encourage them to consider it as a potential career path.
